# Machine learning-based prediction of knee pain risk using lipid metabolism biomarkers: a prospective cohort study from CHARLS

**DOI:** 10.3389/fphys.2025.1607276

**Published:** 2025-06-25

**Authors:** Biao Guo, Yuan Li, Weihang Peng, Yabin Liu, Fei He, Zhe Zhai

**Affiliations:** ^1^ Department of Physical Education, Xi’an University of Posts and Telecommunications, Xi’an, Shaanxi, China; ^2^ Faculty of Health Sciences and Sports, Macao Polytechnic University, Macao, China; ^3^ Faculty of Applied Sciences, Macao Polytechnic University, Macao, China; ^4^ Development Planning Office, Xi’an Physical Education University, Xi’an, Shaanxi, China; ^5^ Sports Humanity and Sociology College, Harbin Sport University, Harbin, Heilongjiang, China

**Keywords:** knee pain, metabolic biomarkers, machine learning, lipid accumulation product, CHARLS

## Abstract

**Introduction:**

Knee pain significantly impairs health and quality of life among middle-aged and older adults. However, the predictive utility of lipid metabolism biomarkers for knee pain risk remains inadequately explored.

**Methods:**

This study utilized data from the China Health and Retirement Longitudinal Study (CHARLS, 2011–2013) to investigate the association between lipid-related metabolic indicators and the risk of knee pain. Multiple lipid biomarkers and composite indices—including the lipid accumulation product (LAP), triglyceride-glucose (TyG) index, and TyG-BMI—were incorporated. Five machine learning models were developed and evaluated for predictive performance. Model interpretation was conducted using SHAP (SHapley Additive exPlanations) to identify the most influential predictors.

**Results:**

A higher prevalence of knee pain was observed in high-altitude, cold regions such as Qinghai and Sichuan provinces. Composite metabolic indices (LAP, TyG, and TyG-BMI) exhibited stronger predictive power than traditional single lipid markers. Among the models, the Stacked Ensemble algorithm achieved the best performance, with an AUC of 0.85 and a Brier score of 0.13. SHAP analysis highlighted LAP and TyG-related indices as the top contributors to prediction outcomes.

**Discussion:**

These findings emphasize the importance of lipid metabolism indicators in the early identification of knee pain risk. The integration of interpretable machine learning approaches and composite metabolic indices offers a promising strategy for personalized prevention in aging populations.

## 1 Introduction

Knee pain is one of the common causes of chronic pain and disability in middle-aged and older adults people. Epidemiological surveys have shown that the prevalence of knee pain increases with age worldwide (Geng et al., 2023; Guo et al., 2023; Liu et al., 2022; Nguyen et al., 2011; Peat et al., 2001). In China, the overall prevalence of knee osteoarthritis (OA), the leading cause of knee pain, is high in middle-aged and older adults people and imposes a heavy burden on individuals and society (Cui et al., 2020). Knee pain often leads to limited activity and decreased quality of life. Because knee osteoarthritis progresses slowly and there is no cure at present (Kim et al., 2011; Wallis et al., 2019), how to identify high-risk individuals at an early stage and take intervention to prevent or delay knee joint degeneration has become the focus of public health and clinical attention (Pelletier et al., 2006; Sharma, 2021). The risk factors for knee pain and osteoarthritis are multifactorial. Recognized risk factors include advanced age, female gender, obesity or overweight (Davis, 1988), and a history of joint overuse or injury. Recent studies have also suggested that insufficient muscle strength, inflammatory state, and genetic susceptibility can increase the risk of knee osteoarthritis (Chapman and Valdes, 2012; Felson, 2004; Musumeci et al., 2015; Valdes and Spector, 2011). However, these factors cannot fully explain the mechanism of knee pain. It is noteworthy that more and more evidence has linked osteoarthritis with metabolic syndrome, and put forward the concept of “metabolic OA” (Hart, 2022; Sekar et al., 2017). The components of metabolic syndrome include obesity, dyslipidemia, hypertension and insulin resistance. Obesity not only acts on the knee through mechanical weight-bearing but also triggers metabolic and inflammatory changes throughout the body, which may directly affect articular cartilage and synovial health (Chadh and a, 2016; Duclos, 2016; Wei et al., 2023). Dyslipidemia (such as high cholesterol and high triglyceride) and insulin resistance related indicators are also reflected in patients with osteoarthritis (Le Clanche et al., 2016; Yan et al., 2024; Yoshimura et al., 2011). Some studies have analyzed NHANES data and found that patients with higher low-density lipoprotein cholesterol (LDL-C) levels have a lower incidence of knee osteoarthritis, suggesting that the relationship between lipid metabolism and joint degeneration is complex (Huang G. et al., 2024; Huang et al., 2024b). Another prospective study reported a 39% increased risk for later arthritis in people with a higher blood triacylglycerol-glucose index (TyG index, a surrogate measure of insulin resistance) (Liu et al., 2024). These evidences suggest that metabolic abnormalities may play a role in osteoarthritis, especially in knee pain, but the specific mechanism and predictive value are still unclear.

Traditional statistical analysis usually studies the association between a single indicator and the occurrence of diseases, which is difficult to fully reveal the nonlinear relationship between the interaction of multiple metabolic factors. Machine learning methods provide a powerful tool for multi-indicator comprehensive analysis, which can mine predictive patterns for disease occurrence from high-dimensional data (Liu et al., 2022; Zhang et al., 2023). In the field of knee pain, some studies have also tried to apply machine learning to construct risk prediction models. For example, Liu et al. used the data of middle-aged and older adults people in the NHANES database to construct a prediction model for the risk of knee pain based on general clinical characteristics and biochemical indicators. The AUC of the logistic regression model was 0.71, and the AUC of the random forest model was about 0.70. The results indicated that waist circumference, BMI, and triglycerides were important predictors in addition to systemic pain (Huang et al., 2024b). Compared with traditional regression models, machine learning models (such as random forests and gradient boosting machines) can more accurately capture the complex relationship between risk factors and outcomes, and provide explanations such as ranking the importance of variables, which is helpful to discover new potential risk factors.

However, the current research on machine learning prediction of knee pain is still limited. In particular, no literature has focused specifically on the predictive value of lipid and metabolism-related biomarkers for the occurrence of knee pain. In the context of the metabolic osteoarthritis hypothesis, it is important to investigate whether lipid metabolism indicators can be used to predict knee pain. On the one hand, it can provide epidemiological evidence for elucidating the role of metabolic factors in osteoarthritis. On the other hand, if the prediction effect is good, blood lipid indicators as a routine clinical test items will help to carry out early screening of patients at high risk of knee pain in primary medical care.

Therefore, this study used data from the China Health and Retirement Longitudinal Study (CHARLS) cohort to select a variety of biomarkers that reflect lipid metabolism status (Zhao et al., 2014), including conventional lipid indicators (TC, TG, HDL-C, LDL-C, non-HDL-C) and combined metabolic index (TyG, TyG-BMI, LAP, CTI). A variety of machine learning methods were used to construct a prediction model of knee pain. CHARLS is a nationally representative cohort of middle-aged and older adults people in China. We will evaluate which markers of lipid metabolism contribute most to the prediction of knee pain by comparing the predictive performance (discrimination and calibration power) of the different models and interpreting the model results by means of variable importance. To enhance the interpretability and clinical trustworthiness of the machine learning models, we further incorporated SHAP (SHapley Additive exPlanations) analysis. SHAP provides both global and individual-level insights into the contribution of each predictor, enabling a transparent understanding of how specific lipid or metabolic biomarkers influence the model’s prediction of knee pain risk. This interpretability is essential in bridging the gap between artificial intelligence applications and practical clinical decision-making, particularly in the early identification and stratification of high-risk individuals (Van den Broeck et al., 2022). Our hypothesis was that certain lipid or metabolic measures (e.g., TyG index or CTI) might have significant predictive effects on knee pain risk and that machine-learning models could improve prediction accuracy. This study is expected to provide a new perspective for understanding the role of lipids in knee health and the early prevention and control of knee pain.

## 2 Materials and methods

### 2.1 Data source and study population

This Study was based on data from the China Health and Retirement Longitudinal Study (CHARLS) in 2011 and 2013. Organized by the Chinese Center for Social Science Survey, Peking University, CHARLS is a national, long-term follow-up survey targeting middle-aged and older adults people aged 45 years and above in China. Using a multi-stage, stratified, cluster random sampling method, the CHARLS covers 28 provinces in China, which has good representability and data quality. The use of data in this study has been approved by the CHARLS data platform and strictly follows its code of ethics for data use. Written informed consent was obtained from all participants. The study has been approved by the Biomedical Ethics Committee of Peking University. The ethical approval number for the part of family questionnaire and physical measurement is IRB00001052-11015, and the ethical approval number for the part of biomarker collection (such as lipid detection) is IRB00001052-11014. Knee pain was determined by self-report in the 2013 follow-up questionnaire: people who answered the question “Do you often feel knee pain in the past 2 years” were considered as new cases of knee pain (Ren et al., 2020).

**FIGURE 1:**
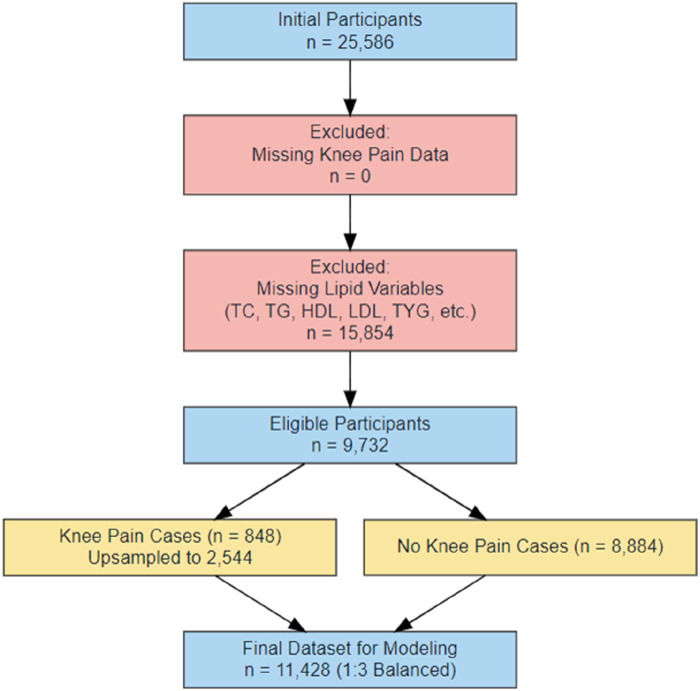
Flow chart of data processing.

In the initial sample, a total of 25,586 respondents were included. We first excluded individuals with missing information on knee pain (n = 0), and then further excluded individuals with missing indicators of lipid metabolism (including total cholesterol (TC), triglyceride (TG), high-density lipoprotein (HDL), low-density lipoprotein (LDL), triglyceride-glucose index (TYG) and its derivatives). A total of 15,854 subjects were excluded. Finally, 9,732 individuals were included for modeling preparation. Among them, 848 patients reported knee pain, which was much lower than that of patients without pain (n = 8,884), and the sample imbalance was significant. To solve the problem of class imbalance, this study used the Synthetic Minority Oversampling Technique (SMOTE) to up-sample the knee pain group to 2,544 cases (Chawla et al., 2002; Fernández et al., 2018; He and Garcia, 2009). By balancing the knee pain group and non-pain group in the final model dataset at a ratio of 1:3, the total number of modeling samples was 11,428. See Figure 1 for details of the data processing flow.

### 2.2 Measurement indicators

At baseline, venous blood samples were collected from participants to measure lipid profiles, including total cholesterol (TC), triglycerides (TG), high-density lipoprotein cholesterol (HDL-C), and low-density lipoprotein cholesterol (LDL-C). Non-HDL cholesterol (NHDL-C) was calculated as TC minus HDL-C. Anthropometric measurements such as height, weight, and waist circumference were also obtained to calculate body mass index (BMI) and various lipid-related metabolic indices.

The triglyceride-glucose (TyG) index was calculated using the formula: TyG = ln [fasting TG (mg/dL) × fasting glucose (mg/dL)/2]. Due to the unavailability of fasting glucose data in CHARLS, we estimated fasting glucose based on glycated hemoglobin (HbA1c), assuming a normal fasting glucose level of approximately 5 mmol/L. The TyG-BMI index was defined as TyG × BMI. The lipid accumulation product (LAP) was calculated as follows: for males, LAP = (waist circumference [cm] − 65) × TG (mmol/L); for females, LAP = (waist circumference −58) × TG. The cholesterol-triglyceride-glucose index (CTI), reflecting the combined level of inflammation and insulin resistance, was computed as CTI = hsCRP (mg/L) × TyG.

Covariates were obtained through structured questionnaires, including sex, age, education level (categorized by academic attainment), marital status (married vs. other), smoking status (current smoker or not), alcohol consumption (frequency in the past year), and residential location (urban or rural).

### 2.3 Model construction

To address the issue of class imbalance, as only approximately 8.7% of participants developed new-onset knee pain in 2013 (n = 848), we applied an oversampling strategy to expand the knee pain cases to 2,544 while retaining all 8,884 pain-free controls. This resulted in a balanced training dataset with a 1:3 case-to-control ratio and a total sample size of 11,428(31–33). The sample selection and data preprocessing procedure are illustrated in Figure 1.

Using the H2O machine learning framework in R, we trained five predictive models on this dataset: generalized linear model (GLM), gradient boosting machine (GBM), random forest (RF), deep neural network (DNN), and a stacked ensemble model (StackedEnsemble). GLM was implemented as logistic regression; GBM and RF were based on decision tree algorithms with boosting and bagging strategies, respectively; DNN was constructed as a multilayer feedforward neural network. The StackedEnsemble model used the predictions of the aforementioned base models as inputs for a meta-learner to enhance overall performance. All models were trained with five-fold cross-validation to tune hyperparameters and reduce the risk of overfitting.

### 2.4 Model evaluation and analysis

In the independent test set, the area under the receiver operating characteristic curve (AUC) was calculated for each model to assess discriminative performance, and ROC curves were plotted for visual comparison (Figure 4). Model calibration was evaluated using the Brier score, with lower values indicating better probability accuracy. Calibration curves (Figure 5) were generated to illustrate the alignment between predicted probabilities and observed outcomes.

To address class imbalance in the outcome variable, the Synthetic Minority Over-sampling Technique (SMOTE) was applied only to the training set after the dataset had been split into training and testing subsets. This design choice avoided information leakage from the test set and ensured a more realistic performance evaluation.

To further evaluate classification performance, confusion matrices were extracted for each model using the optimal threshold determined by the maximum F1-score. From these, we computed sensitivity, specificity, positive predictive value (PPV), and negative predictive value (NPV), which are presented in Table 1 (Visa et al., 2011). Normalized confusion matrix heatmaps were also constructed for each model to intuitively compare misclassification patterns across algorithms (Figure 7) (Susmaga, 2004).

**TABLE 1:** Confusion matrix metrics table.

Model	Sensitivity	Specificity	PPV	NPV
GLM	1	0.006	0.244	1
GBM	0.704	0.799	0.528	0.894
RF	0.719	0.862	0.625	0.905
DNN	0.994	0.012	0.244	0.867
Ensemble	0.663	0.888	0.656	0.892

Note: Values are presented as counts and percentages based on confusion matrix outcomes in the test set. Sensitivity, specificity, positive predictive value (PPV), and negative predictive value (NPV) were computed for each model using the optimal threshold maximizing the F1 score. Accuracy represents the overall classification correctness. DL, deep learning; RF, random forest; GBM, gradient boosting machine; GLM, generalized linear model.

Additionally, variable importance was assessed using the built-in H2O importance metric based on the reduction in model deviance. The top 10 predictors from each model were extracted, and their normalized importance values were aggregated to identify features with consistently high impact across models (Figure 3).

All statistical analyses were conducted using R software version 4.2.2.

### 2.5 Model explainability via SHAP analysis

To enhance the interpretability of the machine learning models and to understand the contribution of individual lipid metabolism biomarkers to knee pain risk prediction, we conducted a comprehensive SHAP (Shapley Additive Explanations) analysis (Sundararajan and Najmi, 2020). SHAP is a unified framework based on cooperative game theory that attributes the contribution of each feature to a model’s output, offering both global and local interpretability. In this study, we used the fastshap and shapviz packages in R (version 4.2.3) to compute and visualize SHAP values for five trained models: generalized linear model (GLM), gradient boosting machine (GBM), random forest (RF), deep neural network (DNN), and stacked ensemble (ENSEMBLE). SHAP values were calculated based on the predicted probabilities for the positive class (i.e., presence of knee pain) extracted from the H2O-trained models. For each model, a prediction wrapper function was defined to extract the “p1” probability, and the fastshap:explain() function was used with 10 Monte Carlo simulations to estimate the SHAP values. To ensure computational efficiency while retaining interpretability, SHAP analysis was performed on a random subset of 50 samples from the test set. All analyses were executed under parallel computation using the do Parallel package with four CPU cores. The computed SHAP values were further visualized using multiple methods provided by shapviz, including bar plots for ranking features by their mean absolute SHAP values, beeswarm plots to depict the magnitude and direction of each feature’s contribution across samples, dependence plots to examine nonlinear interactions between features and SHAP values, and force and waterfall plots for individualized model prediction explanations. The top-ranking features in each model were summarized in the main text, while full sets of SHAP visualizations were presented in Supplementary Material. This interpretability framework allows for a mechanistic understanding of how key lipid-related biomarkers such as LAP, TyG, HDL-C, and CTI influence the predictive decision process across different algorithms (Nohara et al., 2022).

## 3 Results

### 3.1 Baseline characteristics

A total of 25,586 middle-aged and older adults were included in the combined CHARLS 2011–2013 dataset. According to the study design, only individuals with complete data on knee pain assessment and key lipid metabolic indicators—including total cholesterol (TC), triglycerides (TG), high-density lipoprotein cholesterol (HDL-C), low-density lipoprotein cholesterol (LDL-C), triglyceride-glucose index (TyG), fasting plasma glucose (FPG), body mass index (BMI), and waist circumference—were eligible for further analysis. After excluding participants with missing knee pain information (n = 0) and those with missing lipid-related variables (n = 15,854), 9,732 valid samples remained.

Among these, 3,687 individuals with complete covariate data (e.g., marital status, smoking, drinking, and residence) were selected for baseline characteristic analysis to ensure model robustness and avoid bias in the subsequent machine learning process. This subgroup provided a complete dataset with more stable and reliable statistical properties.

Table 2 presents the baseline characteristics of these 3,687 participants, stratified by sex. The overall mean age was 59.32 years (standard deviation: 9.26), with 2,930 males (79.5%) and 757 females (20.5%).

**TABLE 2:** Baseline characteristics of the study population stratified by sex.

Stratified by gender					
Level		Overall	Female	Male	p
n		3,687	757	2,930	
age [mean (SD)]		59.32 (9.26)	59.02 (9.55)	59.40 (9.18)	0.315
cholesterol [mean (SD)]		191.85 (37.58)	199.01 (38.36)	190.00 (37.16)	<0.001
triglyceride [mean (SD)]		128.80 (103.50)	129.43 (84.48)	128.64 (107.89)	0.852
hdl [mean (SD)]		52.72 (16.86)	54.18 (16.24)	52.35 (17.00)	0.008
ldl [mean (SD)]		113.41 (34.57)	119.44 (34.91)	111.86 (34.32)	<0.001
tyg [mean (SD)]		8.65 (0.68)	8.69 (0.62)	8.63 (0.70)	0.045
tyg_bmi [mean (SD)]		204.53 (135.12)	206.27 (40.82)	204.08 (150.15)	0.691
lap [mean (SD)]		2,914.39 (3,661.98)	3,459.06 (3,384.16)	2,773.67 (3,717.99)	<0.001
cti [mean (SD)]		8.73 (0.85)	8.73 (0.83)	8.73 (0.86)	0.979
nhdl [mean (SD)]		76.08 (111.94)	75.25 (93.87)	76.30 (116.16)	0.82
smoking (%)	no	1,226 (33.3)	661 (87.3)	565 (19.3)	<0.001
	yes	2,461 (66.7)	96 (12.7)	2,365 (80.7)	
drinking (%)	yes	3,687 (100.0)	757 (100.0)	2,930 (100.0)	NA
bmi (%)	Normal	2,428 (65.9)	453 (59.8)	1975 (67.4)	0.001
	Obesity	143 (3.9)	41 (5.4)	102 (3.5)	
	Overweight	868 (23.5)	203 (26.8)	665 (22.7)	
	Underweight	248 (6.7)	60 (7.9)	188 (6.4)	
marital_status (%)	married	3,306 (89.7)	633 (83.6)	2,673 (91.2)	<0.001
	single	381 (10.3)	124 (16.4)	257 (8.8)	
residence (%)	Rural	2,439 (66.2)	513 (67.8)	1926 (65.7)	0.312
	Urban	1,248 (33.8)	244 (32.2)	1,004 (34.3)	

Note: Data are presented as mean (standard deviation) for continuous variables and number (percentage) for categorical variables. P-values were calculated using independent t-tests for continuous variables and chi-square tests for categorical variables. HDL: high-density lipoprotein cholesterol; LDL: low-density lipoprotein cholesterol; TyG: triglyceride-glucose index; LAP: lipid accumulation product; CTI: cholesterol-to-triglyceride-glucose index; NHDL: non-HDL, cholesterol; BMI: body mass index.

Regarding lipid indicators, females had significantly higher levels of TC (199.01 vs. 190.00 mmol/L, p < 0.001), HDL-C (54.18 vs. 52.35 mmol/L, p = 0.008), and LDL-C (119.44 vs. 111.86 mmol/L, p < 0.001) than males. No significant sex differences were observed for TG, TyG, TyG-BMI, CTI, or NHDL-C. The lipid accumulation product (LAP), a marker of metabolic obesity, was significantly higher in females (3,459.06 vs. 2,773.67, p < 0.001), indicating greater visceral fat accumulation.

In terms of lifestyle, the smoking rate was markedly higher in males than in females (80.7% vs. 12.7%, p < 0.001), while the drinking rate was uniformly 100% across both sexes. For BMI categories, males had a higher proportion of normal weight (67.4% vs. 59.8%) and overweight (22.7% vs. 26.8%), whereas females showed a higher obesity rate (5.4% vs. 3.5%, p = 0.001). The proportion of married individuals was also significantly higher in males (91.2% vs. 83.6%, p < 0.001). There was no significant difference in urban-rural residence distribution between the sexes (p = 0.312).

These findings suggest that in this middle-aged and older population with a highly imbalanced sex distribution, certain lipid metabolic indicators and sociodemographic variables differ significantly by sex, which warrants consideration and adjustment in further analyses.

### 3.2 Regional distribution of knee pain incidence across provinces

**FIGURE 2:**
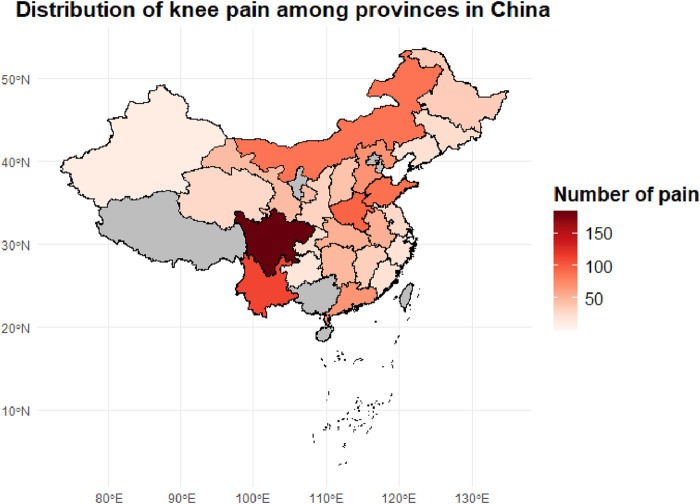
Provincial distribution of knee pain case counts among middle-aged and older adults adults in China. The choropleth map illustrates the number of reported knee pain cases by province based on the CHARLS dataset. Darker shades indicate a higher concentration of knee pain cases, with Sichuan, Hunan, and Henan provinces showing the highest prevalence.

**FIGURE 3:**
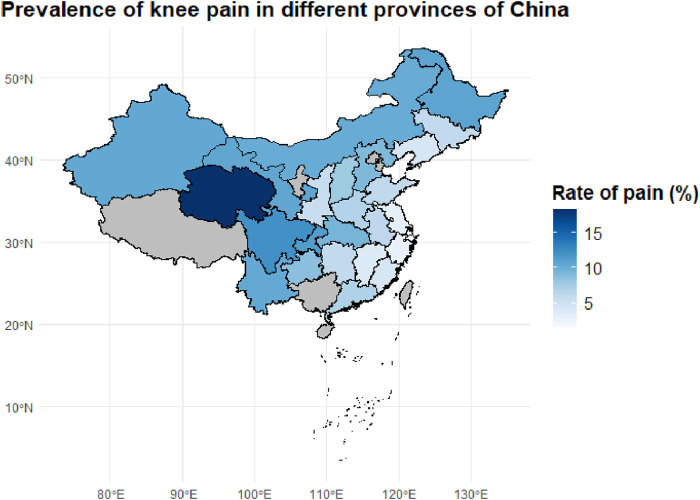
Geographic Distribution of Knee Pain Prevalence Across Chinese Provinces. This map shows the provincial-level prevalence of knee pain among middle-aged and older adults individuals in China, based on the CHARLS dataset. Darker blue areas represent provinces with higher prevalence rates, with Sichuan and adjacent regions showing the highest burden.

To investigate the regional epidemiological characteristics and spatial distribution of knee pain in China, this study conducted a provincial-level aggregation of knee pain cases and their corresponding proportions based on the CHARLS sample. A national distribution map of knee pain case numbers and a heatmap of incidence rates were plotted (Figures 2, 3) to visually present the regional disparities in disease burden.

In terms of case numbers (Figure 2), knee pain cases were mainly concentrated in populous provinces in central and western China, such as Sichuan (184 cases), Yunnan (111 cases), Henan (96 cases), and Shandong (89 cases). These provinces have relatively large sample sizes and high proportions of older adults, constituting the major burden areas for knee pain in the country. These regions are typically characterized by hilly and mountainous terrain, intensive agricultural labor, and moderate to low levels of economic development, which may limit opportunities for regular physical activity and contribute to the higher occurrence of knee pain.

However, case numbers alone do not accurately reflect the actual disease risk in each region. Therefore, we further calculated the proportion of knee pain cases within the provincial samples (i.e., incidence rates), generating a standardized incidence heatmap (Figure 3). The results showed that provinces with the highest incidence rates included Qinghai (18.3%), Sichuan (12.1%), Chongqing (11.3%), Heilongjiang (10.7%), Gansu (10.6%), and Xinjiang (10.4%). These areas are known for their cold climates and are located in high-altitude, high-latitude, or mountainous regions, suggesting that environmental factors such as cold and damp weather may play a significant role in the development of chronic knee pain.

In contrast, southeastern coastal and southern provinces such as Fujian, Zhejiang, and Guangdong exhibited relatively low incidence rates (5%–7%). This may be attributed to their warm and humid climates, better access to healthcare resources, and more diverse physical activity patterns among older adults. The observed spatial heterogeneity suggests that the onset of knee pain is not solely determined by individual-level factors, but is also closely related to geographic, environmental, and lifestyle influences.

### 3.3 Multi-model predictive analysis

To explore the potential of lipid metabolism-related biomarkers in predicting the risk of knee pain, five machine learning models were constructed using a balanced training dataset (n = 11,428; knee pain group: non-pain group = 1:3). These models included Deep Learning (DL), Distributed Random Forest (DRF), Gradient Boosting Machine (GBM), Generalized Linear Model (GLM), and Stacked Ensemble. Multiple evaluation metrics were employed to comprehensively compare the models, including receiver operating characteristic (ROC) curves, area under the curve (AUC), Brier scores, and model calibration curves.

In addition, confusion matrices were generated at the optimal F1-score threshold to assess sensitivity, specificity, and predictive values for each model, providing a clearer view of real-world classification performance. To enhance interpretability, SHAP (SHapley Additive exPlanations) analysis was performed to identify key lipid-related features that contributed most to model predictions. Both global importance rankings and individual-level SHAP visualizations were used to explore the direction and magnitude of each biomarker’s influence. These methods offered deeper insights into the predictive mechanisms of each model and revealed the consistent significance of composite metabolic indices (e.g., LAP, TyG, TyG-BMI) across algorithms.

#### 3.3.1 Variable importance rankings across different models

**FIGURE 4:**
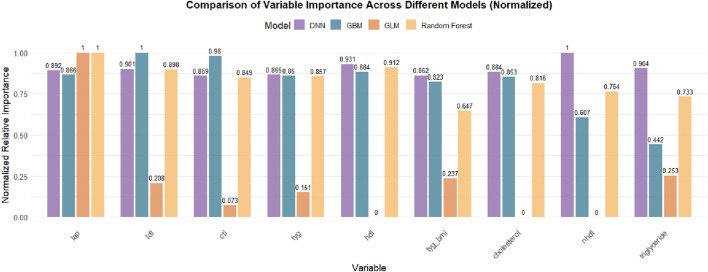
Comparison of Normalized Variable Importance Across Different Machine Learning Models. The bar chart presents the normalized relative importance of each lipid-related biomarker in DNN, GBM, GLM, and Random Forest models. LAP consistently ranked as the most important feature across all models, followed by LDL, CTI, TyG, and HDL, with some variation in the contribution patterns among models. Feature importance values were scaled to the most influential variable within each model.

Figure 4 presents the normalized variable importance rankings for each base model. The lipid accumulation product (LAP) ranked first in all models except for GLM, with a standardized importance of 1.0 in the GBM, DRF, and DL models, indicating its stable and critical contribution to knee pain prediction. Other metabolic indicators such as LDL, HDL, TyG, and TyG-BMI also received relatively high importance scores across several models, reinforcing the predictive value of metabolic obesity and insulin resistance-related markers for chronic joint pain.

It is important to note that the Stacked Ensemble model, by design, integrates the predictions of multiple base learners (e.g., GLM, GBM, DRF, DL) into a two-layer ensemble framework and does not perform variable selection or compute feature importance independently. Therefore, variable importance results for the Stacked Ensemble are not displayed but can be interpreted by referencing the contributing base models. This is a common characteristic of ensemble learning methods, which focus more on improving predictive performance and robustness rather than variable interpretability.

#### 3.3.2 Classification performance of different models (AUC)

To compare the classification performance of the five models, the area under the receiver operating characteristic curve (AUC) was used as the primary evaluation metric. AUC reflects the trade-off between sensitivity (true positive rate) and specificity (1 – false positive rate) across various classification thresholds.

**FIGURE 5:**
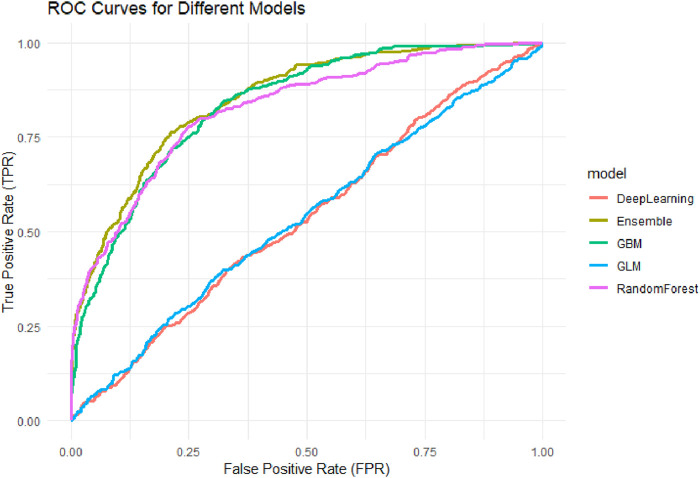
ROC Curves for Different Machine Learning Models. Receiver Operating Characteristic (ROC) curves for five machine learning models: Generalized Linear Model (GLM), Gradient Boosting Machine (GBM), Random Forest (RF), Deep Neural Network (DNN), and Stacked Ensemble. The AUC values of the models were: GLM (0.536), DNN (0.536), GBM (0.835), RF (0.826), and Ensemble (0.850), with the Ensemble model achieving the highest discriminative performance.

As shown in Figure 5, the Stacked Ensemble model demonstrated the best performance with an AUC of 0.85, indicating high accuracy and robustness in distinguishing between positive and negative cases. By integrating multiple base learners (e.g., GBM, DRF, GLM), the model effectively captures both linear and nonlinear patterns, enhancing its overall discrimination ability.

Among the base models, GBM achieved an AUC of 0.83 and DRF 0.82, both showing strong predictive capabilities, particularly in handling complex interactions among variables. The AUC for the GLM model was 0.53, representing a moderate level of performance. This suggests that although GLM offers better interpretability, its ability to model nonlinear and high-dimensional relationships is limited. The DNN model had the lowest AUC at 0.52, potentially due to insufficient model tuning or sparse feature representation, which could undermine its generalizability.

Overall, the ensemble model outperformed the others in AUC evaluation by leveraging multi-model integration, followed by tree-based models (GBM and DRF), while traditional linear regression and neural networks demonstrated relatively weaker adaptability in high-dimensional healthcare data contexts.

#### 3.3.3 Calibration performance of predicted probabilities (Brier score)

**FIGURE 6:**
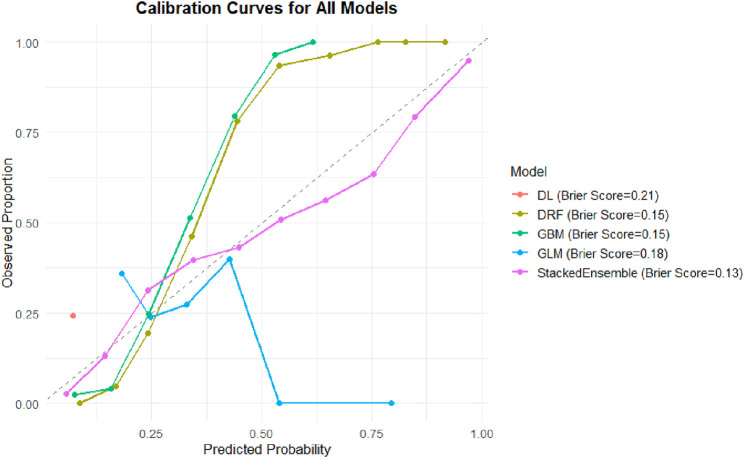
Calibration curves and Brier scores of different machine learning models. Calibration curves comparing predicted probabilities versus observed proportions for five machine learning models: DL, DRF, GBM, GLM, and Stacked Ensemble. The dashed diagonal line represents perfect calibration. The Stacked Ensemble model demonstrated the best overall calibration (Brier Score = 0.13), followed by DRF and GBM (both Brier Score = 0.15), GLM (0.18), and DL (0.21).

In addition to classification performance, the calibration of predicted probabilities is crucial for clinical risk assessment. An ideal classifier should not only differentiate between individuals with and without knee pain but also provide probability estimates that closely reflect the actual likelihood of occurrence. Therefore, this study further evaluated the Brier score and the agreement between predicted and observed probabilities across models (Figure 6).

The Stacked Ensemble model again demonstrated the best performance, with a Brier score of 0.13. Its calibration curve closely followed the ideal 45-degree reference line, indicating excellent calibration across the full range of predicted probabilities. Such strong calibration is essential for designing individualized intervention strategies, particularly in identifying high-risk individuals.

The GBM and DRF models both had Brier scores of 0.15. Although slightly higher than that of the Ensemble model, their predicted probabilities still aligned well with observed proportions, reflecting good calibration in capturing complex variable relationships.

The GLM model had a Brier score of 0.18, with its calibration curve deviating from the reference line in the mid-to-high probability ranges, suggesting under- or overestimation in certain probability intervals. The DL model had the highest Brier score at 0.21, with its calibration curve displaying abrupt shifts and instability in the high-probability region (>0.5), indicating unreliable probability outputs and limited utility for clinical screening.

Notably, while AUC reflects the overall discriminatory ability of a model, the Brier score offers a more accurate measure of “probability fidelity” in public health risk evaluation. From a practical standpoint, the Stacked Ensemble model—with both high AUC and low Brier score—demonstrates superior clinical applicability.

#### 3.3.4 Confusion matrix-based classification performance across models

To enhance the interpretability of model classification results, we extracted confusion matrices at optimal F1-score thresholds and calculated key diagnostic performance metrics, including sensitivity, specificity, positive predictive value (PPV), and negative predictive value (NPV). These metrics are summarized in Table 1.

Among all models, the Stacked Ensemble demonstrated the most balanced performance, with a sensitivity of 66.3%, specificity of 88.9%, PPV of 65.6%, and NPV of 89.1%. This indicates its robust ability to accurately identify both positive (knee pain) and negative (non-pain) cases. The Random Forest model followed closely, with comparable performance (sensitivity = 71.9%, specificity = 86.1%). The GBM model achieved slightly higher sensitivity (70.4%) but showed slightly reduced specificity (79.9%).

In contrast, the GLM and DNN models exhibited skewed prediction behavior. The GLM model classified nearly all cases as positive, resulting in extremely high sensitivity (100%) but extremely low specificity (0.6%), suggesting poor discrimination. Similarly, the DNN model displayed a sensitivity of 99.4% and specificity of only 1.2%, indicating overfitting toward the positive class and lack of generalization.

**FIGURE 7:**
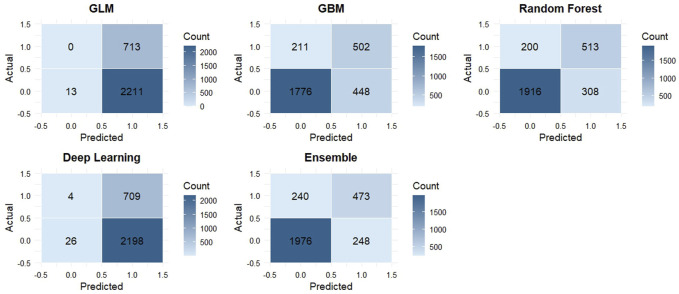
Confusion matrix heatmaps for five machine learning models in the test set. Each panel represents the classification performance of a model: GLM (generalized linear model), GBM (gradient boosting machine), RF (random forest), DL (deep learning), and Ensemble (stacked ensemble). The x-axis shows predicted labels, and the y-axis shows actual labels. Cell values represent the number of observations classified into each category. Darker shades indicate higher counts.

To visually support these findings, we plotted the normalized confusion matrix heatmaps for all models (Figure 7). These heatmaps highlight model-specific misclassification patterns and illustrate the clear performance differences, particularly between tree-based models and linear or neural network models. The superior balance of the Stacked Ensemble’s prediction outcomes supports its reliability for clinical screening applications involving knee pain risk stratification.

#### 3.3.5 SHAP-based feature importance across models

**FIGURE 8:**
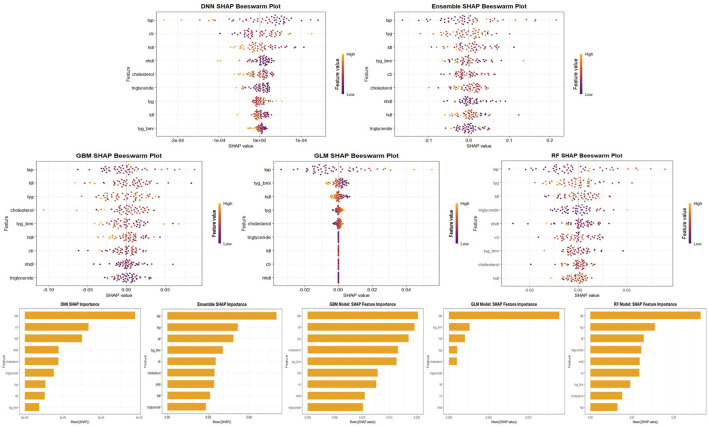
SHAP Feature Importance and Beeswarm Plots Across Models. Visualization of SHAP feature importance and beeswarm plots for five machine learning models (GLM, GBM, RF, DNN, and Stacked Ensemble). The top panel shows SHAP beeswarm plots, revealing the distribution and directionality of feature contributions. The bottom panel shows mean absolute SHAP values for each variable, indicating their global importance. Across all models, LAP, TyG, and TyG-BMI consistently rank among the top predictors of knee pain.

SHAP analysis consistently revealed that composite metabolic indicators were the most influential predictors of knee pain risk across all five machine learning models. Among them, the lipid accumulation product (LAP) emerged as the most important feature, ranking first in nearly all models except the GLM. In the Stacked Ensemble model’s SHAP summary (Figure 8), higher LAP values clearly contributed to increased predicted risk, as indicated by the concentration of high-value points located on the positive side of the SHAP axis. The triglyceride-glucose index (TyG) also ranked among the top predictors, with higher values consistently shifting the model’s output toward greater risk. The TyG-BMI index further reinforced this pattern, emphasizing the joint contribution of dyslipidemia and obesity. In contrast, high-density lipoprotein cholesterol (HDL-C) exhibited a protective effect, with higher values associated with reduced predicted risk, aligning with its known role in cardiovascular and metabolic health.

Although the general hierarchy of feature importance was consistent, some differences emerged among models. Both the GBM and Random Forest models assigned higher weight to low-density lipoprotein cholesterol (LDL-C) and non-HDL cholesterol. SHAP values showed that elevated LDL-C was strongly associated with increased risk, ranking second in the GBM model’s importance scores. In contrast, traditional lipid measures such as total cholesterol and triglycerides were less impactful in the presence of composite indices, suggesting that their predictive information was already captured by aggregated features like TyG and LAP. The GLM model, which demonstrated weaker overall performance, did not emphasize LAP as strongly. The deep learning model (DNN) showed relatively high SHAP values for certain variables, such as CTI, although its predictive accuracy was limited.

Despite these model-specific nuances, LAP, TyG, TyG-BMI, and LDL-C consistently emerged as key predictors across at least four of the five models, while HDL-C demonstrated a consistently negative association with knee pain risk. This convergence reinforces the conclusion that metabolic dysregulation, particularly insulin resistance and central adiposity, plays a critical role in the development of knee pain. Detailed SHAP summaries for each individual model are provided in Supplementary Material, illustrating that the direction and magnitude of each feature’s influence were broadly in line with existing clinical understanding.

## 4 Discussion

This study, based on a large-scale dataset of middle-aged and older adults individuals, analyzed newly developed cases of knee pain. We found that the overall incidence of knee pain is non-negligible and exhibits significant regional disparities: western plateau regions such as Qinghai and Sichuan had a markedly higher prevalence than eastern areas. This pattern suggests that geographic environment and lifestyle may play important roles in the occurrence of knee pain. In terms of predictive factors, we identified a series of metabolism-related indicators—such as lipid accumulation product (LAP), triglyceride-glucose index (TyG), and TyG-BMI—that were closely associated with knee pain risk.

Machine learning models demonstrated that these composite metabolic indicators held high importance and exhibited good predictive performance in identifying individuals at high risk. To further interpret the internal mechanism of the models, we applied SHAP (SHapley Additive exPlanations) analysis to assess the contribution of each feature. The SHAP results consistently highlighted LAP, TyG, and TyG-BMI as the top predictors across multiple algorithms, confirming their dominant role in the prediction process. In contrast, traditional lipid parameters such as HDL-C and total cholesterol showed weaker or even protective effects. These findings not only enhance model transparency but also reinforce the hypothesis that central obesity and insulin resistance-related biomarkers are major contributors to knee joint health deterioration.

Our findings are generally consistent with previously reported epidemiological risk factors for knee disorders, while also providing novel insights. Traditional studies have identified obesity, age, and heavy physical labor as major risk factors for knee osteoarthritis (OA) and knee pain. For example, the prevalence of symptomatic knee OA has been reported to be significantly higher in rural Chinese populations compared to urban counterparts, with physically demanding agricultural labor considered a key contributor to joint degeneration in rural areas (Kang et al., 2009). These findings, derived primarily from traditional statistical approaches such as logistic regression, have offered valuable evidence regarding risk factors for knee disorders. However, such methods often have limited predictive accuracy. A recent review of knee OA risk prediction models pointed out that existing models tend to lack sufficient breadth in the inclusion of risk factors and often fall short in terms of external validation (Ramazanian et al., 2023). In contrast, this study uses machine learning methods (such as random forest) to construct prediction models, which can deal with high-dimensional nonlinear relationships and improve the prediction performance under the comprehensive consideration of multiple factors. In addition, we enriched the range of risk factors for knee pain by introducing composite metabolic measures into the model. This method of combining new indicators and machine learning is rare in similar studies, which reflects the innovation of this study.

In our study, the highest prevalence of knee pain was observed in western provinces such as Qinghai and Sichuan. Based on epidemiological data and literature on metabolic mechanisms, we speculate that this may be attributed to high-altitude environments and regional lifestyle factors. Firstly, chronic exposure to hypoxic conditions at high altitudes may accelerate joint degeneration. Previous studies have demonstrated that long-term residence in plateau regions is associated with decreased bone density and bone strength, which may increase the risk of osteoarthritis (Li et al., 2024). Animal experiments have also shown that simulated hypoxic environments can exacerbate cartilage damage and bone remodeling in knee osteoarthritis models (Li et al., 2024). Physical activity patterns may also differ. While individuals in mountainous areas frequently engage in heavy labor, this form of physical exertion tends to place repeated stress on the knee joints rather than improve joint health. By contrast, urban residents may be more likely to engage in recreational or structured physical activity, which can enhance muscular support and joint function. Access to medical care is another important factor. In many rural or remote areas, limited health resources may delay the diagnosis and treatment of joint-related conditions, leading to higher prevalence and worse outcomes. People living in better-resourced regions may benefit from earlier detection, better management strategies, and preventive interventions, which can reduce the burden of knee pain.

Secondly, the mountainous and plateau terrain in these areas increases the mechanical load on the knee joint during daily activities. A study conducted in rural mountainous regions of Japan reported that overweight individuals living at high altitudes had more than twice the risk of chronic knee pain compared to their normal-weight, low-altitude counterparts (adjusted odds ratio ≈2.13) (Hamano et al., 2014). Qinghai and western Sichuan are predominantly cold, mountainous regions, where residents often engage in strenuous physical labor, such as frequent uphill walking and long-distance travel, due to the complex terrain. These geographical and occupational factors may increase the mechanical stress and wear on the knee joints. In addition, nutritional deficiencies common in these areas, such as vitamin D or selenium deficiency, along with exposure to cold and humid climates, may also contribute to compromised joint health. Some areas have even reported endemic skeletal disorders such as Kashin-Beck disease. Overall, the combination of environmental stressors (e.g., hypoxia and cold temperatures) and heavy physical burden in high-altitude regions may be key contributors to the elevated prevalence of knee pain observed in provinces like Qinghai and Sichuan. This explanation is consistent with the geographic distribution of our data and is further supported by literature documenting a high prevalence of musculoskeletal disorders in high-altitude populations (Li et al., 2024).

This study also emphasizes the mechanistic role of composite metabolic indicators in predicting knee pain. We identified that indices such as LAP (Lipid Accumulation Product), TyG (Triglyceride-Glucose Index), and TyG-BMI—which reflect overall metabolic health—are valuable predictors of knee pain risk. These indicators integrate measures of visceral adiposity and insulin resistance, thereby offering a more comprehensive assessment of metabolic status than single indicators like BMI, fasting glucose, or individual lipid components. A growing body of evidence supports the concept of “metabolic osteoarthritis,” which proposes that metabolic abnormalities contribute to the pathogenesis of osteoarthritis. In obese individuals, adipose tissue secretes pro-inflammatory cytokines, while insulin resistance and hyperinsulinemia may disrupt chondrocyte metabolism and accelerate cartilage degradation (Tchetina et al., 2020). Previous research evidence supports our findings. A study based on data from the National Health and Nutrition Examination Survey (NHANES) reported that LAP is an independent risk factor for osteoarthritis (OA), with a nonlinear increasing trend in OA risk as LAP levels rise. The risk appears to plateau when LAP reaches approximately 120 (cm·mmol/L) (Huang et al., 2024c). Similarly, elevated TyG index levels have been significantly associated with a higher prevalence of knee OA, indicating that individuals with higher TyG scores are more likely to develop the disease (Huang et al., 2024b). It is noteworthy that the TyG-BMI index, which combines the TyG index with body mass index (BMI), demonstrates superior predictive performance. Comparative studies evaluating the diagnostic efficiency of different metabolic indicators for arthritis have shown that TyG-BMI outperforms the standalone TyG index in distinguishing patients with arthritis, with a significantly higher area under the receiver operating characteristic curve (AUC) observed in both Chinese and U.S. populations. This enhanced performance may be attributed to the TyG-BMI index’s ability to simultaneously reflect glucose and lipid metabolism as well as the degree of obesity, making it a more sensitive marker of insulin resistance (Zhang et al., 2024).

Our machine learning results further support the potential mechanisms linking metabolic abnormalities to the development of knee pain. Numerous experimental studies have demonstrated that insulin resistance and hyperlipidemia can trigger chronic low-grade inflammation, leading to the secretion of matrix metalloproteinases (MMPs) by chondrocytes and the subsequent degradation of cartilage extracellular matrix. Elevated levels of low-density lipoprotein cholesterol (LDL-C) may accumulate and oxidize within the synovial membrane, initiating inflammatory cascades that accelerate cartilage degeneration. Correspondingly, epidemiological studies have observed that the use of statins may reduce the risk of knee osteoarthritis (OA), suggesting a possible protective effect of lipid-lowering therapy on joint health. In contrast, high-density lipoprotein cholesterol (HDL-C) exerts anti-inflammatory and antioxidant effects, capable of neutralizing LDL-induced inflammatory responses (Tang et al., 2016). Fang et al. (2024) analyzed data from the NHANES cohort and found that individuals with higher HDL-C levels had a significantly lower incidence of knee osteoarthritis (OA) (Fang et al., 2024). Consistently, our results also demonstrated a negative association between HDL-C and knee pain in machine learning models, supporting its potential protective effect. Collectively, these findings suggest that metabolic factors may influence knee joint health through inflammation-mediated and cartilage metabolic pathways. This study emphasizes the importance of managing lipid profiles and improving insulin sensitivity as part of the primary prevention strategies for knee joint disorders. Taken together, composite metabolic indices such as LAP, TyG, and TyG-BMI may serve as mechanistic indicators reflecting underlying metabolic disturbances and chronic inflammation, playing a critical role in the development of knee pain. The predictive value of these indicators has been validated in several high-quality studies, and our findings further extend their application to musculoskeletal health research.

This study carries significant public health implications. Globally, hundreds of millions of people suffer from osteoarthritis (OA), and this number is rapidly increasing (Zhang et al., 2024). As an early clinical manifestation of OA, knee pain deserves heightened attention. Since no current treatments can reverse OA progression, prevention and early intervention remain the most effective strategies (Geng et al., 2023; Mora et al., 2018). Our findings confirm that several metabolic indicators are predictive of knee pain risk, suggesting that metabolic health assessments should be integrated into musculoskeletal health management for middle-aged and older adults. For instance, incorporating indices such as LAP or TyG—calculated from waist circumference, blood lipids, and glucose—into routine community health screenings could help identify individuals with metabolic abnormalities who are at high risk for knee joint disorders. Once identified, targeted interventions such as weight loss, dietary modification, muscle strengthening, and metabolic control can be implemented to reduce the risk of knee pain and OA development.

In particular, for high-incidence areas identified in this study, enhanced health education and early preventive measures are essential to alleviate the burden of knee joint disorders among populations in plateau and rural regions. Furthermore, the robust predictive performance demonstrated by our machine learning models highlights the promising role of artificial intelligence in public health screening. By constructing multifactorial prediction tools, health authorities can more effectively allocate resources and direct limited interventions toward those most in need. In summary, this study provides new insights and evidence for early screening and intervention of knee pain and underscores the importance of managing metabolic health in maintaining musculoskeletal function among aging populations—contributing meaningfully to efforts aimed at mitigating the growing burden of osteoarthritis worldwide.

In addition to assessing prediction accuracy, our study emphasized model interpretability through SHAP (Shapley Additive Explanations) analysis, which provided detailed insights into how each metabolic biomarker influenced the model’s predictions of knee pain risk. The use of SHAP value decomposition allowed us to not only identify which lipid variables were most important, but also to understand the direction and magnitude of their impact at the individual level. This contributes to a more nuanced understanding of the metabolic underpinnings of knee pain beyond traditional regression-based associations.

Across all five machine learning models, SHAP consistently identified LAP (Lipid Accumulation Product), TyG (Triglyceride-glucose index), and TyG-BMI as the top predictors positively associated with increased risk of knee pain. These composite indices reflect central adiposity, insulin resistance, and dyslipidemia, and have been increasingly recognized as indicators of systemic metabolic stress. Our SHAP-based interpretation supports this view by showing that high values of these indices not only appeared among the top-ranked predictors but also yielded high positive SHAP values, meaning they directly increased the predicted probability of knee pain. This aligns with emerging concepts of “metabolic osteoarthritis” and provides mechanistic plausibility to our predictive findings.

Conversely, HDL-C (high-density lipoprotein cholesterol) consistently demonstrated negative SHAP values, suggesting a protective effect. This is biologically plausible given HDL’s known anti-inflammatory and chondroprotective properties. The consistency of SHAP rankings across models strengthens the generalizability of these findings and demonstrates that ensemble and tree-based models are capturing biologically meaningful relationships rather than spurious correlations.

The interpretability afforded by SHAP is particularly valuable for clinical translation. Unlike traditional black-box models, SHAP allows clinicians to understand why a particular patient is flagged as high-risk—for example, due to an elevated LAP or TyG index. This feature-level attribution can support personalized intervention planning, such as recommending targeted metabolic control in patients with high LAP or low HDL-C to mitigate the risk of developing knee pain.

Furthermore, the ability to visualize SHAP effects (via bar plots, beeswarm plots, and dependence plots) facilitates transparent communication of model behavior, which is crucial for integrating machine learning into real-world decision-making in aging and musculoskeletal health. These visualizations, included in the main text (Figure 8) and (Supplementary Material), enable reproducibility, regulatory review, and clinical trust.

In summary, SHAP analysis validated the predictive significance of composite lipid-related indices and highlighted their biological roles in knee pain development. This not only enhances the explainability of our models but also bridges predictive modeling with mechanistic understanding, laying a foundation for future intervention studies targeting metabolic pathways in osteoarthritis prevention.

This study has several limitations. First, this study was based on the CHARLS dataset, and external validation was not feasible due to the lack of comparable nationwide cohorts with consistent biomarker profiles. While internal cross-validation and an independent test set were used to assess robustness, the generalizability of the model should be further verified in future studies using external datasets. knee pain was assessed via self-reported questionnaire, which introduces a degree of subjectivity and potential for underreporting or misclassification. However, the question targeted frequent and persistent pain (“Have you often experienced knee pain in the past 2 years?”) (Ren et al., 2020), which likely captured moderate to severe cases with reasonable validity. Second, the CHARLS dataset lacks imaging data, preventing us from distinguishing whether the reported knee pain was attributable to radiographically confirmed knee osteoarthritis (KOA). Therefore, our model predicts symptomatic knee pain rather than the incidence of radiographic KOA *per se*. Nevertheless, from a public health perspective, knee pain alone holds clinical relevance and impacts quality of life. Future studies integrating imaging assessments are warranted to further evaluate the predictive value of metabolic markers for structural joint changes. This study did not include covariates such as comorbid diabetes, or rheumatoid arthritis due to data limitations. These factors are potential confounders and may influence both lipid metabolism and knee pain outcomes. Additionally, the outcome variable—knee pain—was self-reported and thus subject to recall bias and misclassification. Future studies incorporating clinical diagnoses, objective biomarkers, and activity monitoring are warranted to further validate these findings.

## 5 Conclusion

Based on the nationally representative CHARLS cohort, this study systematically evaluated the association between lipid metabolism-related biomarkers and knee pain risk among middle-aged and older adults individuals in China. The findings reveal that composite metabolic indicators—such as LAP, TyG, and TyG-BMI—exhibited significantly better predictive power than traditional single lipid markers. Among all machine learning approaches applied, the stacked ensemble model demonstrated the highest performance in both discrimination and calibration.

To enhance model transparency, we introduced SHAP (SHapley Additive exPlanations) analysis, which provided individualized and global interpretability of prediction results. The SHAP bar and beeswarm plots consistently identified LAP and TyG as the most influential predictors across models, highlighting their central role in knee pain pathogenesis. This interpretability not only validates the robustness of machine learning predictions but also strengthens their practical value in clinical decision-making and screening strategies.

In addition, we observed a markedly higher prevalence of knee pain in high-altitude, colder western provinces of China. This geographic pattern suggests a potential interplay between environmental stress, lifestyle, and metabolic abnormalities in contributing to joint degeneration. Together, these findings offer novel insights into the metabolic basis of knee pain and emphasize the value of explainable AI for early identification, precision prevention, and targeted interventions in high-risk populations.

## Data Availability

The datasets presented in this study can be found in online repositories. The names of the repository/repositories and accession number(s) can be found below: Publicly available datasets were analyzed in this study. This data can be found here: http://charls.pku.edu.cn/.

## Abbreviations

CHARLS, China Health and Retirement Longitudinal Study; LAP, Lipid Accumulation Product; TyG, Triglyceride-Glucose Index; TyG-BMI, TyG × Body Mass Index; CTI, C-Reactive Protein-Triglyceride-Glucose Index; BMI, Body Mass Index; HDL-C, High-Density Lipoprotein Cholesterol; LDL-C, Low-Density Lipoprotein Cholesterol; NHDL-C, Non-High-Density Lipoprotein Cholesterol; GLM, Generalized Linear Model; GBM, Gradient Boosting Machine; DRF, Distributed Random Forest; DNN, Deep Neural Network; AUC, Area Under the Receiver Operating Characteristic Curve; ROC, Receiver Operating Characteristic; OA, Osteoarthritis; KOA, Knee Osteoarthritis; hsCRP, High-Sensitivity C-Reactive Protein; SMOTE, Synthetic Minority Oversampling Technique; MMP, Matrix Metalloproteinase; NHANES, National Health and Nutrition Examination Survey.
