# Supplementary material for: Machine learning-based prediction of knee pain risk using lipid metabolism biomarkers: a prospective cohort study from CHARLS
Source: Front Physiol. 2025 Jun 25;16:1607276. doi: 10.3389/fphys.2025.1607276 (PMC12239094; doi:10.3389/fphys.2025.1607276)
Supplement: Supplementary file 1 [file DataSheet1.docx]

Supplementary Material

## Supplementary Figures


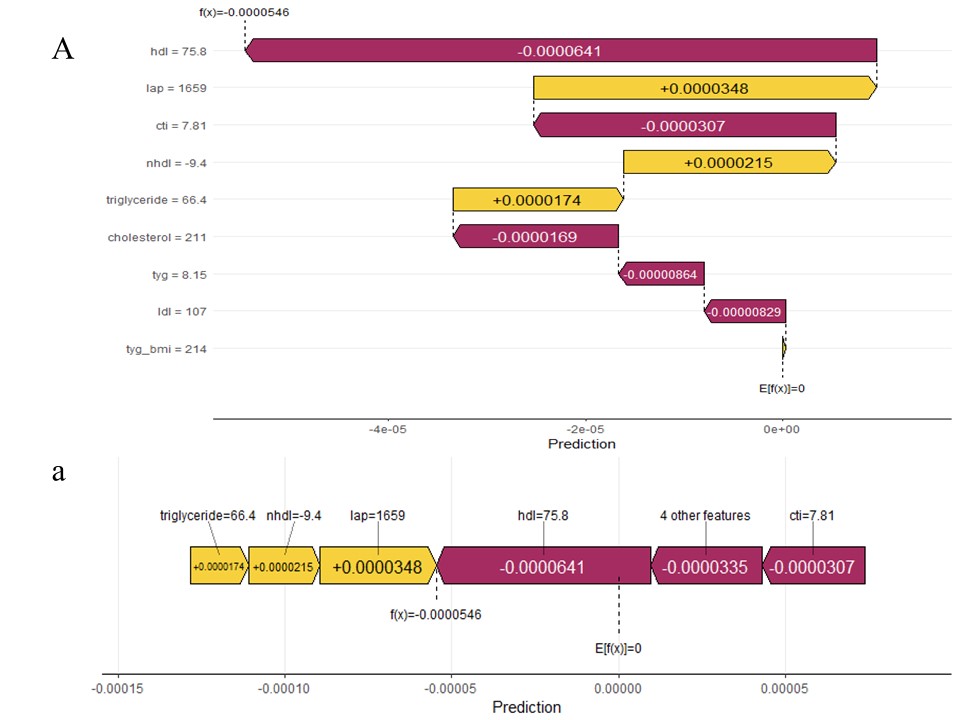


**Supplementary Figure 1.** SHAP visualization for an individual prediction using the deep neural network (DNN) model. The top panel illustrates a SHAP force plot, where each bar represents the contribution of a specific variable to the final prediction. Features that increase the predicted probability of knee pain are shown in yellow (positive SHAP values), while those that decrease the prediction are shown in purple (negative SHAP values). The length of each bar indicates the magnitude of the contribution. The bottom panel presents a SHAP decision plot for the same case, tracing the cumulative contribution of each feature along the prediction path from the model’s expected value to the final predicted value. This individual-level explanation highlights the roles of triglycerides, LAP, HDL-C, and CTI in influencing risk, supporting the relevance of composite metabolic indicators in the prediction of knee pain.


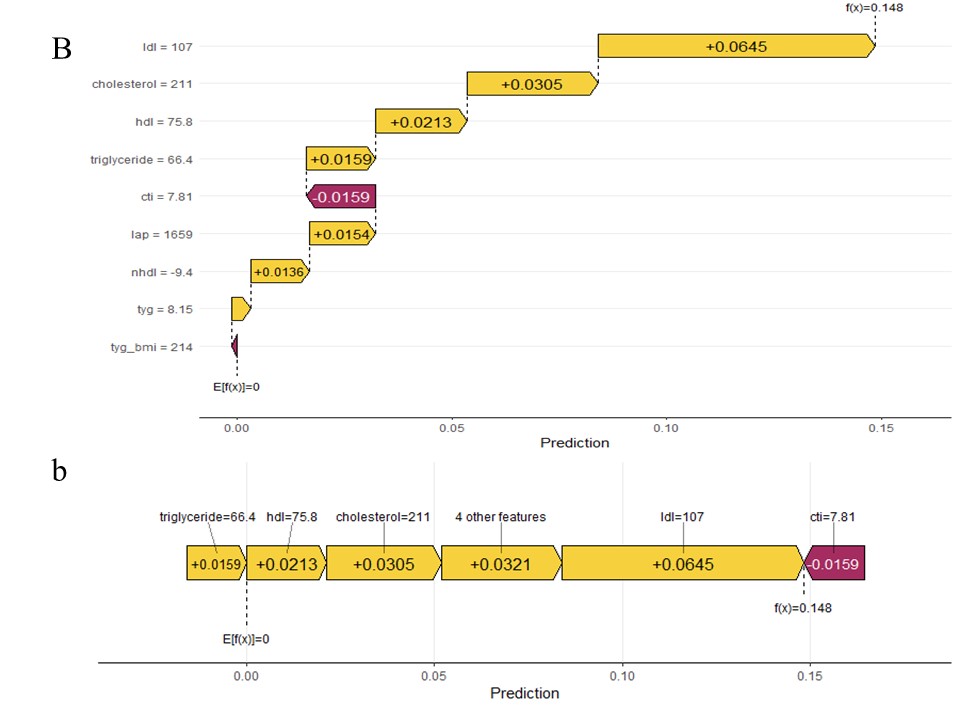


**Supplementary Figure 2.** SHAP-based explanation of an individual prediction generated by the stacked ensemble model. The upper panel displays the SHAP force plot, where each feature’s contribution to the predicted knee pain risk is visualized through arrows—yellow indicates positive contributions and purple indicates negative ones. The cholesterol and LDL-C levels had the most significant positive impact on increasing the prediction, while CTI slightly decreased the predicted risk. The lower panel shows the SHAP decision plot for the same individual, tracing how the cumulative effects of each variable sequentially build from the baseline prediction (E[f(x)] = 0) to the final output probability (f(x) = 0.148). This visualization provides clear interpretability of how different lipid-related variables interact to influence the model’s decision for this participant.


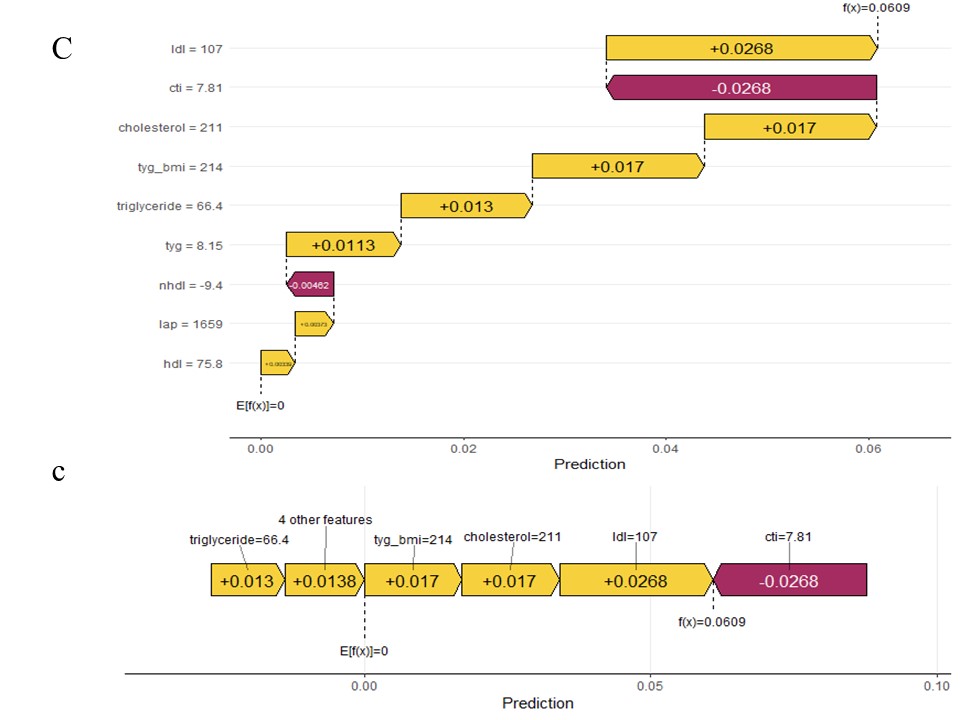


**Supplementary Figure 3.** SHAP visualization for another individual prediction made by the stacked ensemble model. The top panel displays the SHAP force plot, showing both positive and negative contributions of individual lipid biomarkers to the predicted knee pain risk. LDL-C and cholesterol exerted the most prominent positive impact, increasing the final prediction probability, while CTI made an equally strong negative contribution, effectively balancing the prediction. The bottom panel shows the SHAP decision plot for the same case, demonstrating the additive influence of each feature from the model’s base value (E[f(x)] = 0) to the final predicted probability of 0.0609. This figure illustrates the model’s reasoning process and supports the interpretability of SHAP in individualized risk assessment.
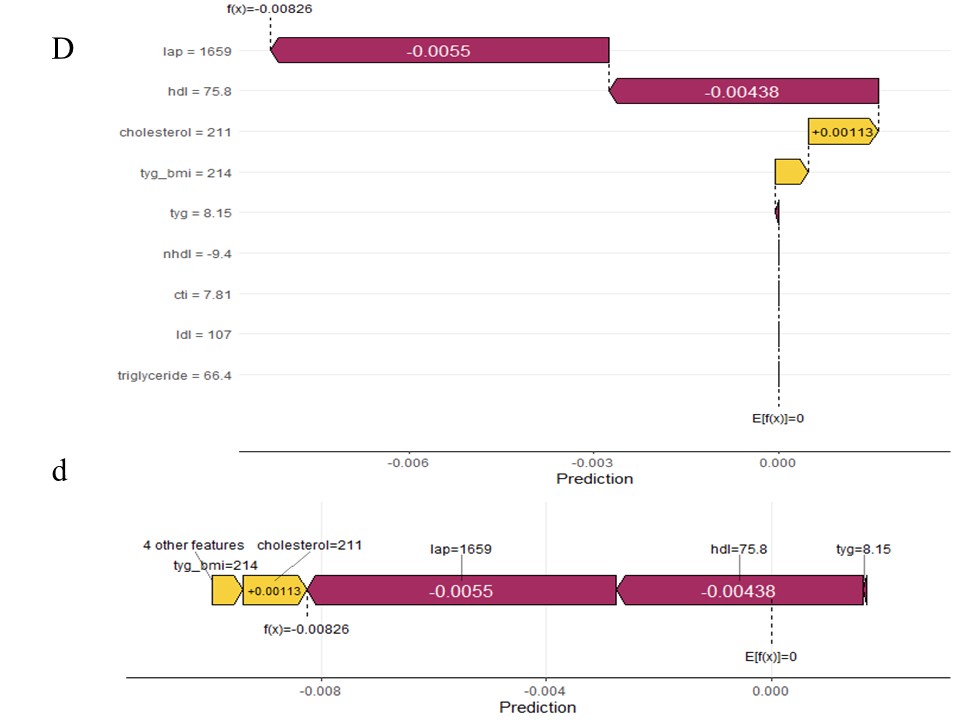


**Supplementary Figure 4.** SHAP visualization for an individual prediction made by the stacked ensemble model showing a negative predicted knee pain risk. The top force plot demonstrates that the major contributing factors to the low predicted risk were LAP and HDL-C, both exerting substantial negative SHAP values that reduced the final probability. A small positive contribution from TyG-BMI was not sufficient to reverse the prediction. The bottom decision plot presents the cumulative SHAP contributions from all features, tracing how the model’s prediction shifts from the base value to the final output of −0.00826. This case exemplifies how protective metabolic features can jointly lower the model’s risk estimate.


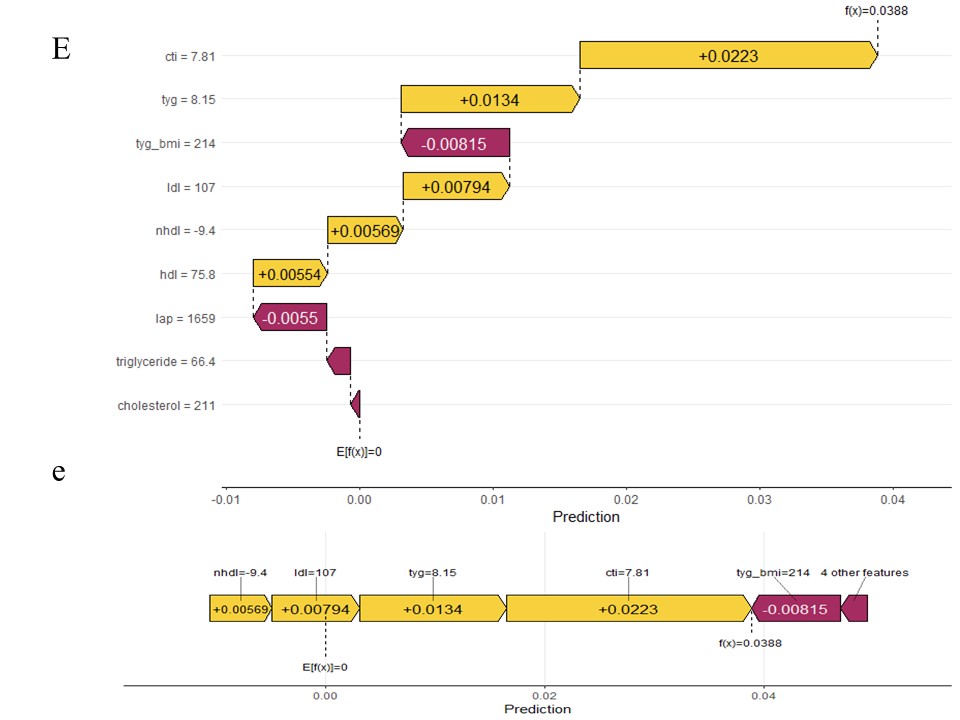


**Supplementary Figure 5.** SHAP visualization for an individual prediction generated by the stacked ensemble model with a moderate positive knee pain risk prediction (f(x) = 0.0388). The top force plot illustrates that CTI and TyG were the strongest positive contributors to the predicted risk, while TyG-BMI and LAP exerted negative effects. The bottom decision plot shows the sequential contribution of each feature from the model’s base value to the final prediction, highlighting that despite negative SHAP values from LAP and composite lipid indices, the risk remains elevated due to higher values in predictors such as CTI, TyG, and LDL-C. This example demonstrates the balance between protective and risk-enhancing features in shaping the model’s final output.


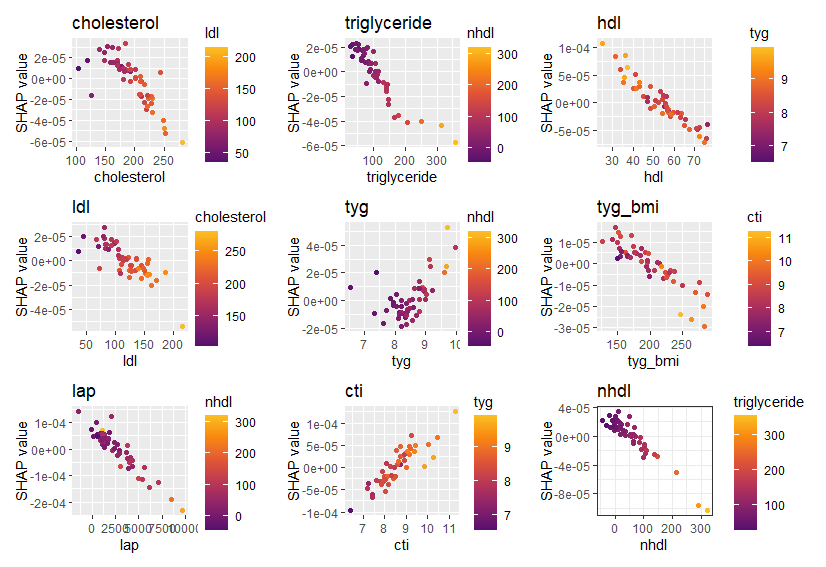


**Supplementary Figure 6.** SHAP dependence plots with color-coded interactions for each lipid-related predictor in the Stacked Ensemble model. Each subplot shows the SHAP value of a given feature on the y-axis and its corresponding value on the x-axis, while point color indicates the value of the strongest interacting feature. For example, the effect of triglyceride is modulated by non-HDL cholesterol, as indicated by the color gradient, suggesting a synergistic effect. Similarly, the impact of TyG on predicted risk varies depending on non-HDL levels, and LAP interacts significantly with non-HDL as well. These interaction patterns reveal complex nonlinear relationships among lipid biomarkers in the model, further supporting the relevance of composite metabolic indicators in predicting knee pain risk.


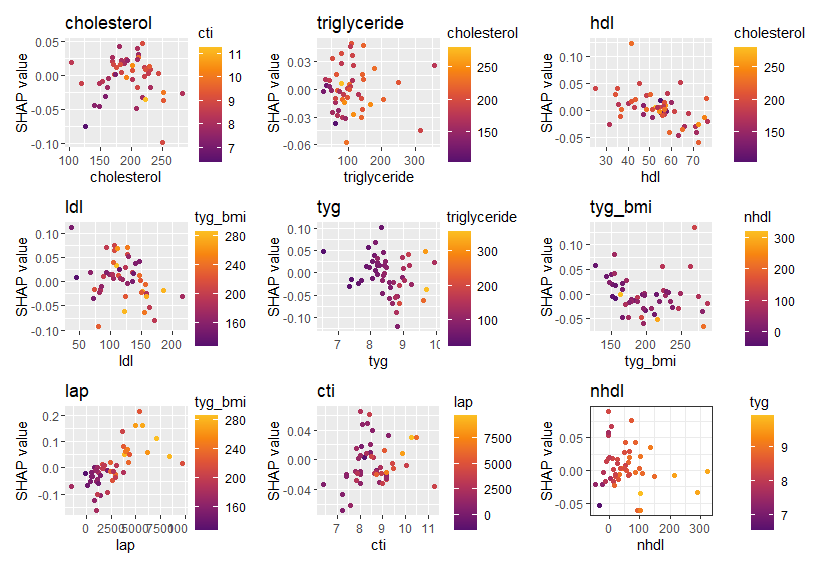


**Supplementary Figure 7.** SHAP dependence plots of lipid metabolism-related features under the Stacked Ensemble model. Each subplot illustrates the relationship between the SHAP value of a specific feature and its original value, with color gradients representing the value of another interacting feature. The plots highlight second-order interactions that modulate the effect of individual predictors on the model output. For example, the SHAP impact of triglyceride is influenced by cholesterol, while the effect of LAP is modulated by TyG-BMI. These results reveal that complex interactions among lipid markers contribute significantly to the ensemble model’s prediction of knee pain risk.

**
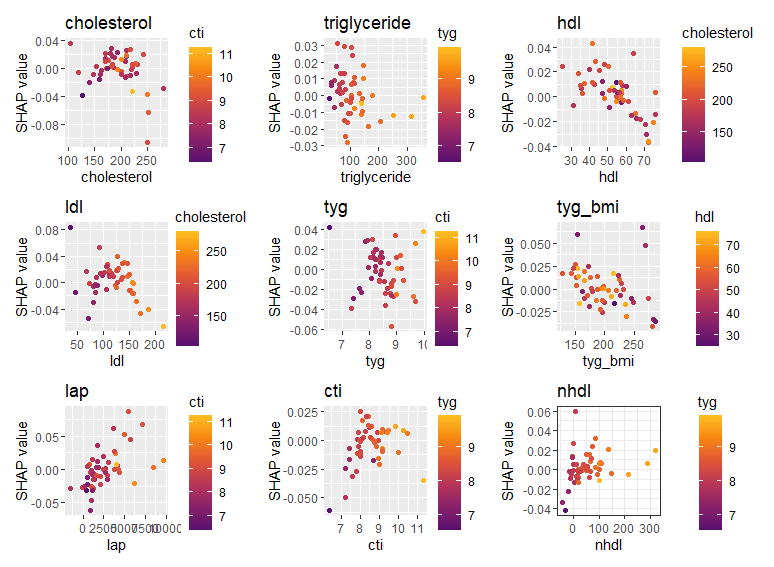
**

**Supplementary Figure 8.** SHAP dependence plots for each lipid-related biomarker in the GBM model. Each subplot shows the SHAP value on the y-axis, representing the contribution of a given feature to the model’s prediction for knee pain, plotted against the actual value of the feature on the x-axis. Color gradients indicate the interaction effects with another biomarker (e.g., TyG, CTI, cholesterol, etc.), highlighting potential synergistic or antagonistic relationships. The observed nonlinear patterns suggest complex interactions between individual lipid indices and model output, particularly for composite metabolic indicators like LAP, TyG, and TyG-BMI.

**
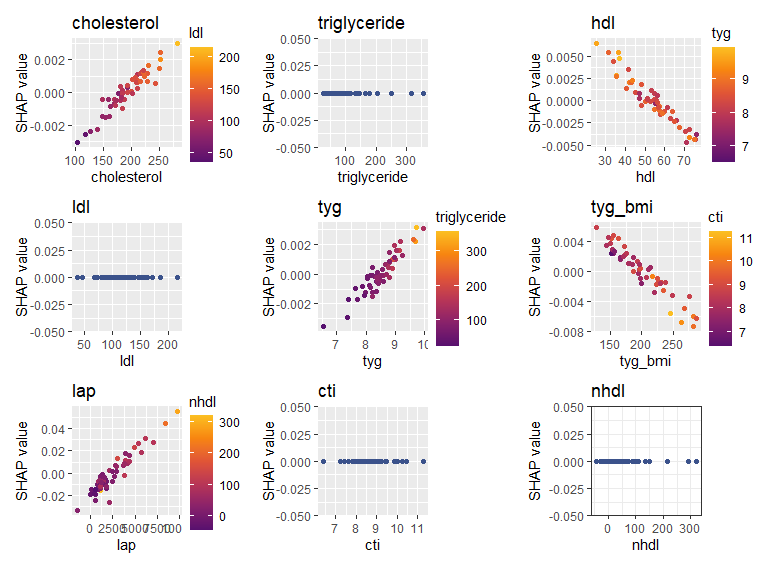
**

**Supplementary Figure 7.** SHAP dependence plots for each lipid-related biomarker in the GLM model. Each panel displays the relationship between the actual value of a feature (x-axis) and its corresponding SHAP value (y-axis), reflecting the feature’s contribution to the model’s prediction for knee pain. Colored gradients indicate interactions with another biomarker. Compared to nonlinear models, most GLM SHAP values exhibit limited variation, reflecting the model’s inherent linear structure. Notably, features like LAP, TyG, and HDL still show directional effects, aligning with their known metabolic roles.

**
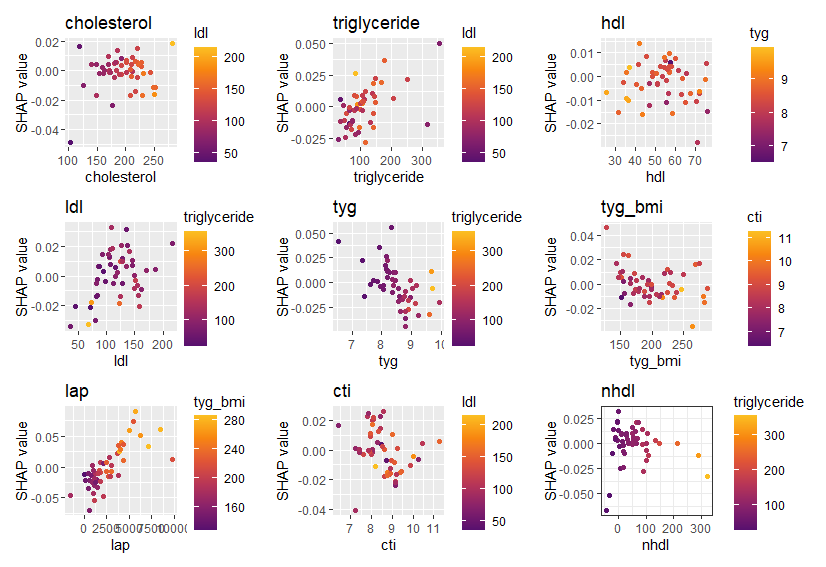
**

**Supplementary Figure 10.** SHAP dependence plots for the Random Forest model, showing how each lipid-related variable contributes to the prediction of knee pain across different ranges. The x-axis represents the original value of the feature, while the y-axis shows the corresponding SHAP value, indicating its influence on the model's prediction. Colored gradients reflect interactions with another selected variable. Notably, LAP, TyG, and TyG-BMI show clear positive associations with SHAP values, suggesting their strong contribution to elevated risk. Conversely, HDL demonstrates a negative association, consistent with its protective role in metabolic health.
